# Long-term avian influenza virus epidemiology in a small Spanish wetland ecosystem is driven by the breeding Anseriformes community

**DOI:** 10.1186/s13567-019-0623-5

**Published:** 2019-01-17

**Authors:** Olalla Torrontegi, Vega Alvarez, Pelayo Acevedo, Xeider Gerrikagoitia, Ursula Höfle, Marta Barral

**Affiliations:** 1 Animal Health Department, NEIKER-Instituto Vasco de Investigación y Desarrollo Agrario, Parque tecnológico de Bizkaia P-812, 48160 Derio, Bizkaia Spain; 2 Grupo SaBio, Instituto de Investigación en Recursos Cinegéticos, IREC (CSIC-UCLM-JCCM), Ronda de Toledo 12, 13071 Ciudad Real, Spain

## Abstract

During 2007–2009 and 2012–2014, avian influenza virus (AIV) was studied in a wild avian community of a northern Spanish wetland using non-invasive sampling methods and host identification by *COI* barcoding. The aim of this longitudinal study was to evaluate AIV dynamics in a natural wetland ecosystem, taking into account both virological aspects and ecological traits of hosts. Global AIV prevalence decreased significantly during the second sampling period (0.3%) compared to the first (6.6%). Circulating subtype distributions were also different between periods, with a noteworthy H5 and H7 subtype richness during the first sampling period. Mallard *Anas platyrhynchos* was identified as the main AIV host, although not all positive samples could be ascribed to the host. We modelled AIV prevalence with regard to the avian host community composition and meteorological data from the wetland. Statistical analysis revealed seasonal differences in AIV detection, with higher prevalence during the breeding season compared to other phenological events. The model also shows that the lower AIV prevalence during the second study period was associated with a significant reduction of breeding Anseriformes in the wetland, revealing a long-term fluctuation of AIV prevalence driven by the breeding Anseriformes community. This longitudinal study on AIV epidemiology in a natural ecosystem reveals that although prevalence follows seasonal and annual patterns, long-term prevalence fluctuation is linked to the breeding community composition and size. These results are relevant to understanding the influence of host ecology on pathogen transmission for preventing and managing influenza emergence.

**Electronic supplementary material:**

The online version of this article (10.1186/s13567-019-0623-5) contains supplementary material, which is available to authorized users.

## Introduction

Avian influenza viruses (AIV; family *Orthomyxoviridae*, genus *Influenzavirus A*) owe their diversity to the antigenic properties of two surface glycoproteins [16 known haemagglutinin variants (HA) and 9 neuraminidases (NA)], that combined lead to 144 potential HA/NA subtypes [1]. Although most AIV subtypes are low pathogenic (LPAIV) to wild birds and poultry, H5 and H7 subtypes can become highly pathogenic (HPAIV) after infecting domestic gallinaceous birds [2, 3]. Occasionally, some AIV strains may also become zoonotic [4]. Therefore, also LPAIV need to be under stringent surveillance as they pose a risk to both animal and public health. The Asian A/goose/Guangdong/1/96 HPAIV H5N1 is a good example of geographical dispersal and interspecies transmission capacity of AIV. Until 2005, it was considered an Asian phenomenon, but in 2006 it expanded westwards reaching Europe and Africa. Ever since, HPAIV H5N1 has been present in more than 60 countries, fatally affecting birds with occasional spillovers to mammals (humans included) and its circulation still continues to the present day [5]. More recently, in 2014, an Asian HPAIV H5N1-derived HPAIV H5N8 emerged in Europe and it was thought to cause only mild disease in wild birds [5]. The HPAIV H5N8 strain concurrently spread in Siberia, South Korea, Japan and the United States [6] and by August 2017 at least 1112 HP H5N8 outbreaks in poultry and 955 in wild birds had been detected in 30 European countries, the largest epizootic detected in the continent [7]. Most H5N8 outbreaks in domestic and wild birds were small, of the size of 30 km radius, possibly linked between them, and it is believed that wild birds have played a role in the introduction of AIV into Europe [7].

The avian orders Anseriformes and Charadriiformes are considered the natural reservoirs of AIV [8]. Most known subtypes have been found in species of these orders in which infections tend to occur in the absence of clinical signs [9, 10]. Particularly, the presence of Anseriformes is considered essential for AIV transmission and environmental persistence [2]. Among all the Anseriformes taxa, the mallard (*Anas platyrhynchos*) is the most well studied species and seems to have a key role in AIV epidemiology in natural settings in the Northern Hemisphere [11]. Mallards harbour the highest AIV subtype diversity, prevalence is usually high and most viral isolations are recovered from this taxon [10]. However, introduction of AIV can also occur occasionally by other (spillover) hosts, making ecological approaches at the wild bird community level fundamental in AIV epidemiology [12, 13].

AIV prevalence in wild ecosystems is dynamic and dependent on a wide variety of factors such as time of the year, environment, location, circulating subtype and infected host species [2, 14–17]. Epizootics in Europe are related to seasonal patterns of host species [18] and tend to be more frequent in late summer/early autumn, albeit varying in time with latitude [8, 19]. While seasonal patterns appear consistent, significant interannual variations exist [18, 20]. Within the Anseriformes, AIV prevalence has been associated with premigrational staging and high concentrations of juveniles (with up to 30% of infection rates within this age group), presumably due to their naïve immune system [8, 19]. Some authors have noted that once in wintering areas, prevalence rapidly decreases, probably as a consequence of acquired herd immunity [21–23]. Heterosubtypic immunity also seems to influence the prevalence and dynamics of different AIV subtypes in hosts such as the mallard [24]. Wintering individuals have demonstrated to be able to amplify locally circulating AIV and phylogeography has shown AIV to switch from intraspecific to interspecific transmission in wintering quarters, probably as part of the AIV persistence strategy [18, 22, 25]. Transmission between hosts fundamentally occurs through the faecal-oral route after shedding AIV infected particles in faeces to water bodies, with considerable variation in environmental persistence between subtypes [19, 26]. For this reason, long-term studies are of special interest when conducting AIV monitoring in aquatic ecosystems. Yet, the existing studies are still scarce and very heterogeneous [14, 17, 20, 27–29]; while some aim a single avian order (or even taxon), others have been conducted during specific periods of the year, or focused on different sampling locations sometimes with low representative samplings [10, 17, 20, 30, 31]. Consequently, comparable epidemiological results from natural environments are difficult to obtain. Given the relatively high AIV prevalence previously found in the Salburua wetland [2], a better understanding on AIV dynamics and epidemiology will only be achieved by long-term studies which consider the local wild avian host community and its ecology.

The aim of this longitudinal study was to evaluate AIV dynamics in a natural wetland ecosystem, by taking into account virological aspects and ecological traits of hosts during two different sampling periods. Our sampling strategy was based on non-invasive sampling methods, which consisted of environmental fresh faecal sample collection after *de visu* identification of host and flushing [and further host identification by mitochondrial cytochrome oxidase subunit 1 (*COI*) barcoding]. Hence, all roosting waterbird species were regarded as potential hosts and interspecies natural mixing was considered. Special attention was paid to circulating viral strains, to host species harbouring AIV and to ecological factors with a potential effect on viral detection.

## Materials and methods

### Study area

Salburua wetland (42°51′N 002°39′W; altitude 500–510 m) is a 217.46 ha wetland area located in Basque Country that pertains to the “Northern-Plateau” bioregion of Spain [32]. Under the Atlantic climate influence, Salburua wetland has a noteworthy thermal oscillation along the year with dry summers (infrequent precipitation, reduced relative humidity and high diurnal temperatures). Annual mean temperature is 11.4 °C (5.1 °C in winter and 17.9 °C in summer, with large temperature differences between day and night in this season) and the annual mean precipitation is 823.4 mm. The number of ground frost days is moderate (40.8 days/year) [33].

Salburua wetland is composed of various lagoons surrounded by meadows and a small oak grove. It received the *Wetland of International Importance* Ramsar designation in 2002 and *Site of Community Importance* in 2004 within the European Natura 2000 Network. From an ornithological perspective, the wetland is strategically situated, as many bird species use these lagoons for wintering, breeding or for stopover along the East Atlantic flyway while migrating. The bird community on Salburua wetland includes mallards, coots (*Fulica atra*), Northern shovelers (*Anas clypeata*), white storks (*Ciconia ciconia*), gadwalls (*Mareca strepera*), common teals (*Anas crecca*), common pochards (*Aythya ferina*), common moorhens (*Gallinula chloropus*) crested grebes (*Podiceps cristatus*) and little grebes (*Tachybaptus ruficollis*), with varying numbers depending on the season, especially for migratory species. Since 2005, when monitoring plans for AIV began in the Basque Country, this wetland has had a frequent LPAIV record and the only HPAIV H5N1 wild bird case in the Iberian Peninsula so far [2, 12].

### Sample collection

Collection of fresh avian faeces started at dawn, at roosting sites of waterbirds that included nine scattered islets in the wetland and seven wet meadow areas, covering almost all the available surface used by waterbirds. The number of places sampled each month was determined by the flood level of the lagoons. The species that most frequently used these roosting areas were various species of ducks, geese, storks, grey herons, coots, gulls and Eurasian spoonbills, generally as mixed flocks of varying size throughout the year. In order to minimise the risk of sampling the same bird more than once, we were careful to collect only recently deposited droppings, placing the complete dropping in an individual sterile container. Samples were kept refrigerated until analysis within the next 24 h. As the study wetland had been part of a comparative study on ecological drivers of AIV prevalence in different wetlands [2], faecal samples, AIV isolates and bird census data were still available for the former study period (2007–2009). We submitted the available material to complementary analysis (virus subtyping and host identification), obtaining a dataset to compare host population and AIV specific data, between a former period of high LPAIV prevalence with a latter period of significantly lower prevalence. In the former period, samplings were performed once every three months during 2007–2009, with a total of 667 samples obtained from eight sampling visits, from which 44 were AIV-positive (6.6%) (for details see [2]). As for the latter sampling period, a total of 2725 samples were collected from March 2012 until September 2014 during 31 monthly sampling visits.

### AIV detection

Four to five individual faecal samples were pooled according to species (when possible), appearance and location from where they were found. Viral RNA extraction was performed with a commercial kit (RNeasy Mini Kit, Qiagen, Hilden, Germany) following the manufacturer’s instructions. Samples were screened with a TaqMan™ (ThermoFisher Scientific Inc., Waltham, USA) real time reverse transcription polymerase chain reaction (rRT-PCR) for AIV matrix-gene detection [34]. Amplification was carried out using AgPath-ID™ One-Step RT-PCR Reagents (ThermoFisher Scientific Inc., Waltham, USA).

At detection of an AIV-positive pool, a new individual RNA extraction from the pool-composing units was performed in an attempt to identify the positive sample by rRT-PCR.

The same viral detection procedure and reagents were used for both sampling periods.

### AIV isolation

AIV isolation was performed from rRT-PCR positive samples, including individual faecal samples or pools when the AIV-positive unit could not be identified. Approximately 25 mg of the original faecal samples were homogenised with 500 µL of Hank’s Balanced Salt Solution supplemented with penicillin (2000 μ/mL), streptomycin (2 mg/mL) and sodium bicarbonate 7.5%, final pH 7.0–7.4 (ThermoFisher Scientific Inc., Waltham, USA). The mixture was inoculated into the allantoic cavity of five embryonated specific pathogen free (SPF) eggs after 9–11 days of incubation according to standard procedures [35]. Viral RNA extraction was carried out by incubation at 58 °C for 1–3 h, 180 µL of the allantoic fluid, 2.7 µL carrier RNA (1 µg/µL) and 20 µL Proteinase K (20 mg/mL) followed by extraction in a Biosprint 96 robot with a DNA Blood kit (Qiagen, Hilden, Germany) following the manufacturer’s instructions. Samples were analysed for the presence of AIV matrix gene by rRT-PCR [34]. In cases where no AIV was isolated, the harvested allantoic fluid was reinoculated into a new set of SPF eggs and processed as previously described.

### AIV subtype identification and pathogenicity

HA and NA were determined from both faecal RNA and isolates either by rRT-PCR [34, 36–39]. Pathogenicity of the H5- and H7-positive samples was determined by the haemagglutinin cleavage site sequencing [40–42].

### Host identification

Host identification was only attempted for AIV positive faecal samples. DNA was extracted from these samples using MagMAX™ Total Nucleic Acid Isolation Kit (ThermoFisher Scientific Inc., Waltham, USA) according to the manufacturer’s instructions. It consisted of a nested PCR targeting the *COI* gene. AWCF1 and AWCR6 primers were used for the first round and AWCintF4 and AWCintR6 for the second round [43]. Alternatively, first round External F1 and External R1 [44] primers were used combined with the former second round primers. The 277 bp amplified PCR fragments were purified using Illustra™ ExoProStar™1-Step (GE Healthcare Europe, Freiburg, Germany) according to the manufacturer’s instructions, and were sequenced in an AB3130 Genetic Analyzer (ThermoFisher Scientific Inc., Waltham, USA), using BigDye^®^ Terminator v3.1 Cycle (ThermoFisher Scientific Inc., Waltham, USA). The obtained sequences were compared with those published on the network server of the National Centre for Biotechnology Information with BLAST^®^.

### Longitudinal epidemiological analysis

#### Ecological data

Wetland authorities provided monthly data of bird species’ abundance from the whole wetland area. Each of the censuses was performed by the same wetland officer within a single day in order to minimise bias related to observer skills or repeated counts. Counts were performed early in the morning, but not at dawn to ensure to include individuals spending the night elsewhere (mainly *A. platyrhynchos*). The counting method used consisted of a series of fixed observing points, from which the observer counted all relevant birds in a sector of the lagoon. For that end, 8 × 30 binoculars and a 20 × 60 magnifying telescope were used. Data of waterbird counts were grouped (Table 1) according to the following: species taxonomy; taxonomic implication on AIV epidemiology (Anseriformes and Charadriiformes reservoir hosts or not); interspecies feeding associations (grazers, gulls and divers), bird species pertaining to the Anatinae subfamily were also grouped according to surface water or diving feeding behaviour (dabbling ducks vs. diving ducks). In parallel, waterfowl phenological traits in the area (wintering season: November–January; northward spring migration: February–April; breeding season: May–July and southward autumn migration: August–October) [45] were taken into account for species categorization. In this regard, counted species were labelled as resident, winterer, breeder or migrant (depending on species more than one category could be assigned, as an example, in mallards in addition to the resident local population many individuals, mostly from central, but also from northern Europe are present during the wintering period) according to local information [46, 47] and L. Lobo’s personal communications. During 2010 and 2011 bird censuses were performed but no samples for AIV detection were taken. During breeding season, an additional census was performed by the same wetland officer as in the monthly censuses. This encompassed the whole wetland area and consisted of nest counting to infer the minimum number of breeding couples at the Salburua wetland. Daily meteorological parameters from sampling day, 7 and 15 days before sampling (bs) were obtained from the Basque Meteorological Agency (Euskalmet) (Table 2) and added to the dataset [48].

**Table 1:** **List of predictors related to the avian community inhabiting the Salburua wetland used for building the model**

Predictor	Definition
Census of wild birds	Total wild bird counts per census session (monthly)
Species richness	Number of wild bird species
Order	Counts of waterbird per orders (Anseriformes, Charadriiformes, Ciconiiformes, Gruiformes, Pelecaniformes, Podicipediformes)
Species	Counts of waterbird per species
Anseriformes and Charadriiformes	Counts of Anseriformes and Charadriiformes individuals
non-Anseriformes and non-Charadriiformes	Counts of non-Anseriformes and non-Charadriiformes individuals
Anatini	Counts of dabbling ducks
Aythyini	Counts of diving ducks
Grazers	Counts of *Anas penelope*, *Anas crecca*, *Anser anser* and *Fulica atra*
Gulls	Counts of *Larus michahellis*, *Larus fuscus* and *Chroicocephalus ridibundus*
Divers	Counts of *Aythya ferina*, *Aythya fuligula and Fulica atra*
Phenology	Waterfowl life cycle events: northward spring migration, breeding, southward autumn migration, wintering^a^
Breeding couples	Counts of breeding couples per species (*Anas clypeata*, *Anas platyrhynchos*, *Anas strepera*, *Anser anser*, *Aythya ferina*, *Aythya fuligula*, *Ardea cinerea*, *Ardea purpurea*, *Ciconia ciconia*, *Circus aeruginosus*, *Charadrius dubius*, *Fulica atra*, *Gallinula chloropus*, *Himantopus himantopus*, *Ixobrychus minutus*, *Larus michahellis*, *Nycticorax nycticorax*, *Podiceps cristatus*, *Rallus aquaticus* and *Tachybaptus ruficollis*)
Breeding Anseriformes	Counts of breeding couples of Anseriformes members
Breeding non-Anseriformes and non-Charadriiformes	Counts of all breeding couples excluding Anseriformes and Charadriiformes
Summer visitor birds	Counts of summer visitor birds
Summer visitor species richness	Number of summer visitor species
Winter visitor birds	Counts of winter visitor birds
Winter visitor species richness	Number of winter visitor species
Migratory birds	Counts of migratory birds
Migratory species richness	Number of migratory species
Resident birds	Counts of resident birds
Resident species richness	Number of resident species

VIF < 2 in underline.

^a^Wintering: November–January; northward spring migration: February–April; breeding: May–July and AM southward autumn migration: August–October.

**Table 2:** **List of predictors related to the meteorological data that were used for building the model**

Predictor	Definition
Mean temperature (°C)	Sampling day, 7 days bs and 15 days bs
Maximum temperature (°C)	Sampling day, 7 days bs and 15 days bs
Minimum temperature (°C)	Sampling day, 7 days bs and 15 days bs
Total precipitation (l/m^2^)	*Sampling day*, 7 days bs and *15* *days bs*
Mean humidity (%)	*Sampling day*, *7* *days bs* and 15 days bs
Mean wind (km/h)	Sampling day, 7 days bs and *15* *days bs*
Maximum gust of wind (km/h)	*Sampling day*, 7 days bs and 15 days bs

VIF < 2 in italics.

bs: before sampling.

#### Statistical analysis

We used the number of AIV-positive samples at each sampling visit (35 sampling visits; 8 for 2007–2009 and 27 for 2012–2014) in relation to sample sizes as the response variable. A generalized linear model (GLM) (binomial distribution, logit link function) was used to assess the effects of ecological factors (namely, phenology, bird counts and climate; see below) explaining variations in AIV positivity in this longitudinal study. In addition to the aforementioned variables, predictors previously described (Tables 1 and 2) were included as covariables. We avoided multicollinearity derived problems using the variance inflation factor (VIF); covariables with VIF > 2 were not considered for modelling [49]. VIFs were calculated for each variable as the inverse of the coefficient of non-determination of the regression of each predictor against all others using the R package “HH” [50]. The variables selected after controlling the VIF were considered in the GLM. The final model was obtained using a forward–backward stepwise procedure based on the corrected Akaike Information Criteria to compare models. The Post-hoc Tukey’s test was performed to assess for differences between pairs of phenological periods. Differences were considered significant when *p* < 0.05.

## Results

### AIV prevalence and subtype richness

A total of 2725 faecal samples were collected during 2012–2014. Global AIV prevalence was 0.3% (8/2725), which was significantly lower when compared to the 2007–2009 period 6.6% (44/667) (GLMz: Z = − 8.04, *p* < 0.001) (Table 3). Sampling effort and AIV prevalence detected at each sampling time are detailed in an additional file (Additional file 1). Viral recovery-rate from both sampling periods was 48% (25 virus isolations out of the 52 AIV positive records). AIV isolations were only achieved from samples taken during autumn migration (Table 4).

**Table 3:** **Yearly prevalence of AIV detected and bird phenological event**

Year	Spring migration		Breeding		Autumn migration		Wintering		Total	
	Prev (%) ± 95% CI	Pos/N	Prev (%) ± 95% CI	Pos/N	Prev (%) ± 95% CI	Pos/N	Prev (%) ± 95% CI	Pos/N	Prev (%) ± 95% CI	Pos/N
2007	–	–	–	–	–	–	0.0	0/95	0.0	0/95
2008	0.0	0/154	31.4 ± 16.2	11/35	2.1 ± 4.3	1/47	0.0	0/136	3.2 ± 1.8	12/372
2009	1.0 ± 2.0	1/98	18.2 ± 27.2	2/11	31.9 ± 9.7	29/91	–	–	16.0 ± 5.1	32/200
Total 2007–2009	0.4 ± 0.8	1/252	28.3 ± 13.5	13/46	21.7 ± 7.0	30/138	0.0	0/231	6.6 ± 1.9	44/667
2012	0.0	0/214	1.0 ± 1.5	2/192	0.0	0/102	0.0	0/203	0.3 ± 0.4	2/711
2013	0.0	0/305	0.0	0/106	0.9 ± 0.8	4/465	0.3 ± 0.5	1/384	0.4 ± 0.3	5/1260
2014	0.0	0/159	0.3 ± 0.6	1/341	0.0	0/129	0.0	0/125	0.1 ± 0.3	1/754
Total 2012–2014	0.0	0/678	0.5 ± 0.5	3/639	0.6 ± 0.5	4/696	0.1 ± 0.3	1/712	0.3 ± 0.2	8/2725

– Indicates not sampled.

Prev: AIV prevalence, CI: 95% confidence interval, N: number of samples analysed, Pos: number of AIV positive samples.

**Table 4:** **Distribution of AIV subtypes in Salburua according to waterbird phenology, year, isolation and host species**

Year	Phenology	N	Isolation N	Subtype	Identified host (N)
2008	BR	11	8	H3N8	*Anas platyrhynchos* (7), ND (4)
	AM	1	0	H5N2	*Anas platyrhynchos* (1)
2009	SM	1	0	ND	ND (1)
	BR	2	0	ND	ND (2)
	AM	1	1	H4N?	ND (1)
	AM	1	0	H6N5	ND (1)
	AM	2	2	H7N2	*Anas platyrhynchos* (1), ND (1)
	AM	1	0	H7N8	*Anas platyrhynchos* (1)
	AM	1	1	H7N9	*Anas platyrhynchos* (1)
	AM	4	1	H7N?	*Anas platyrhynchos* (2), ND (2)
	AM	4	3	H11N2	ND (4)
	AM	9	6	H11N9	*Anas platyrhynchos* (4), ND (5)
	AM	3	0	H11N?	*Anas platyrhynchos* (2), ND (1)
	AM	1	1	H7/H11; N4/N9^a^	ND (1)
	AM	2	0	ND	*Anas platyrhynchos* (1), ND (1)
Total 2008–2009		44	23		20
2012	BR	1	0	H3N8	*Anser anser* (1)
	BR	1	0	ND	ND^b^ (1)
2013	AM	1	1	H3N2	*Anas platyrhynchos* (1)
	AM	1	1	H3N8	ND (1)
	AM	1	0	H12N5	*Anas platyrhynchos* (1)
	AM	1	0	ND	ND (1)
	W	1	0	H5N?	*Anas platyrhynchos* (1)
2014	BR	1	0	ND	*Anser anser* (1)
Total 2012–2014		8	2		5
Total		52	25		25

AM: southward autumn migration, BR: breeding season, SM: northward spring migration, W: wintering season, N: number of samples, ND: not determined, ?: not identified.

^a^A mixed infection, it was not possible to elucidate what haemagglutinin type corresponded to its respective neuraminidase.

^b^This sample pertains to a pool from 3 *Fulica atra* and 1 *Anser anser*, but whose positive unit was not possible to determine.

Considering both sampling periods, 2007–2009 and 2012–2014, 11 different viral subtypes were identified (Table 4). H3N8 was the most frequent subtype (25% of all AIV-positive samples, 13/52) followed by H11N9 (17%, 9/52). A high diversity of circulating low pathogenic H5 (PQRETR*GLF) and H7 (PEIPKGR*GLF) strains was found (Table 4).

Host identification was successful in 48% (25/52) of the AIV-positive samples (Table 4). All identified host species were anatids [*Anas platyrhynchos*: 44% (23/52); and *Anser anser*: 3.8% (2/52)].

#### Longitudinal study

**Figure 1:**
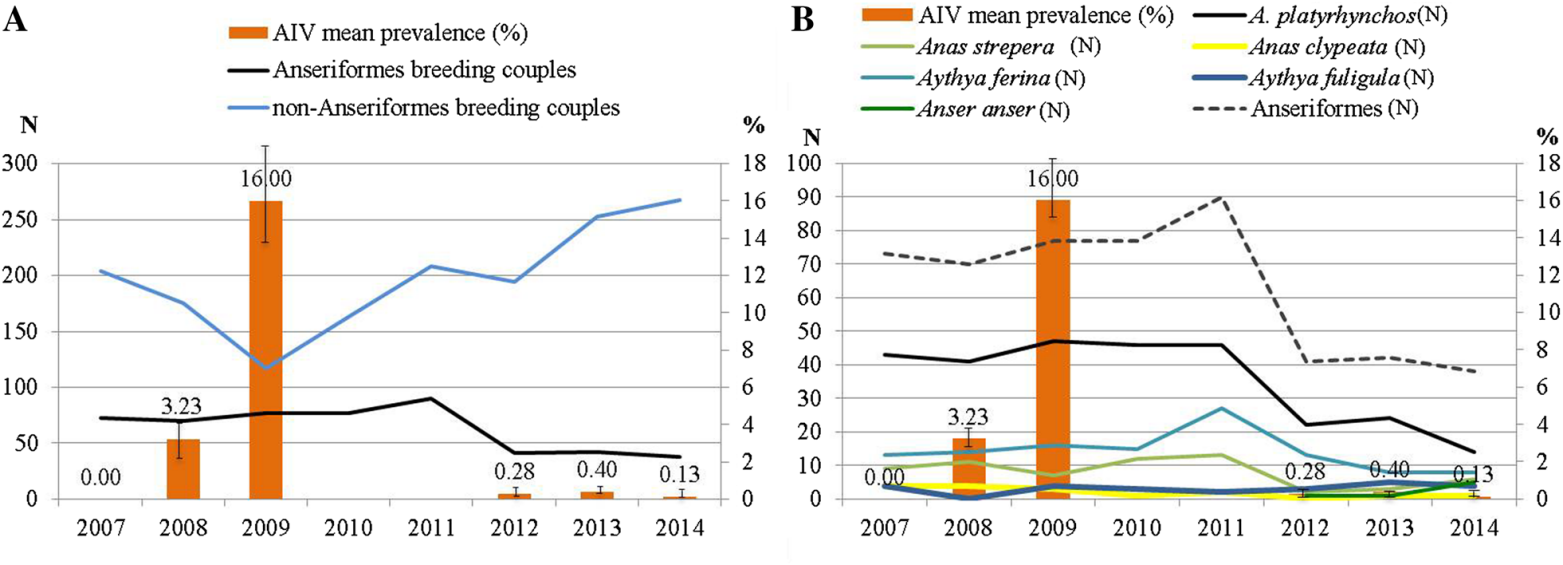
**Mean AIV yearly prevalence and counts of Anseriformes and non-Anseriformes breeding pairs. A** Total counts of Anseriformes vs. non-Anseriformes breeding pairs. **B** Total counts of species of Anseriformes breeding pairs. During 2010 and 2011 breeding pairs were counted but no samples for AIV detection were taken.

**Figure 2:**
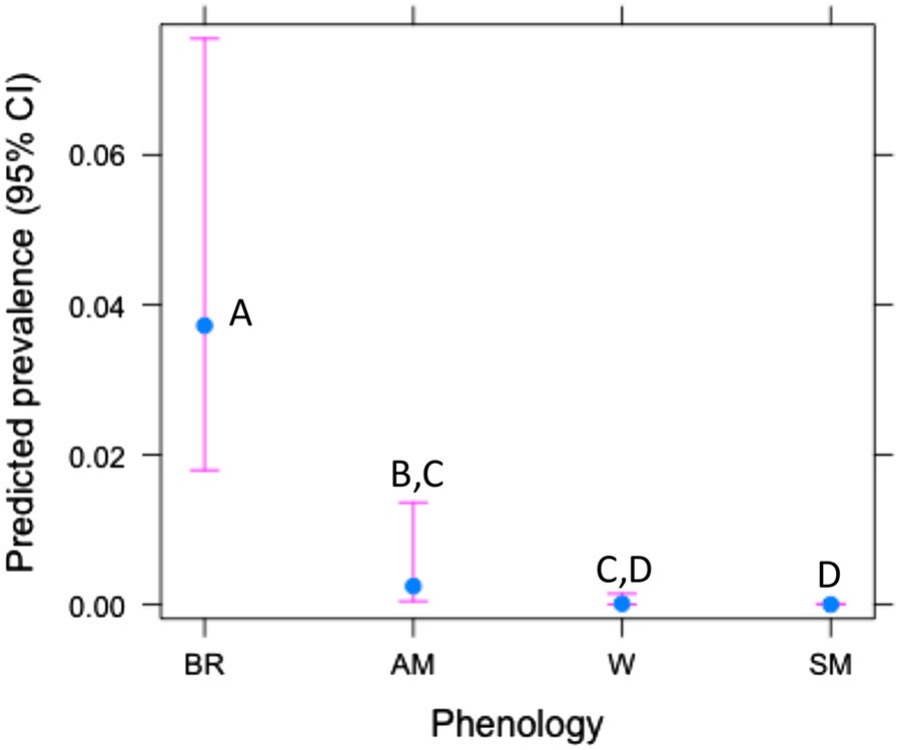
**Predicted AIV prevalence in relation to phenology.** *BR* breeding season, *AM* southward autumn migration, *W* wintering, *SM* northward spring migration. Means sharing the same letter did not differ significantly (Tukey tests *p* > 0.05).

After VIF analysis, the following variables were selected for modelling: number of migratory species, resident species richness, summer visitor species richness, number of breeding moorhen couples (*Gallinula chloropus*), breeding grebe couples (*Podiceps cristatus*), breeding little grebe couples (*Tachybaptus ruficollis*), breeding Anseriformes, and breeding non-Anseriformes/non-Charadriiformes (Table 1 and Figure 1). Mean humidity of sampling day and 7 days bs, total precipitation of sampling day and 15 days bs, maximum gust of wind of sampling day, mean wind 15 days bs, and phenology were also considered for building our model (Tables 1, 2 and Additional file 2). The final model explains 95.7% of the total deviance. The results indicate a strong positive relation between AIV prevalence and the number of Anseriformes breeding couples (see Table 5). There was also a positive relation with wind during the 15 days bs, with resident species richness and with breeding season. In addition to these variables, the Tukey test showed significant differences in AIV prevalence according to waterbird phenology; namely higher prevalence rates during breeding season followed by autumn migration, while AIV was less prevalent during wintering season and spring migration (with no significant differences between autumn migration and wintering neither between wintering and spring migration periods) (Figure 2).

**Table 5:** Variables in the final model for AIV prevalence, their coefficients, statistical test value and significance

	Estimate	SE	z value	Pr(> |z|)
(Intercept)	−37.586	7.832	−4.799	***
Breeding Anseriformes couples	0.140	0.024	5.934	***
Phenology				
Breeding	2.755	1.018	2.706	**
Spring migration	−6.862	1.307	−5.252	***
Wintering	− 3.195	1.522	−2.099	*
Resident species richness	1.111	0.316	3.521	***
Mean wind 15 days bs (km/h)	0.965	0.282	3.425	***
Breeding *Podiceps cristatus* couples	−0.193	0.090	−2.140	*

Coefficients for phenology are relative to southward autumn migration.

SE: standard error, bs: before sampling

***p < 0.001; **p < 0.01; *p < 0.05.

## Discussion

Our study provides information on the relation between long-term AIV prevalence variation and a number of ecological and environmental drivers (including Anseriformes host demography) in a small wetland in northern Spain and adds to the still scarce information on long-term AIV dynamics. The present work is a continuation to a short-term cross-sectional study that characterised several different wetlands in parallel. That study, compared factors that modulated LPAIV prevalence in a particular wetland and showed that location and climate are more important than (but also interact with) the size and composition of the waterfowl community [2]. Here, we used the wetland that showed the highest LPAIV prevalence in the aforementioned study, to more closely explore the interaction between LPAIV and avian host ecology, as well as the influence of the latter on long-term LPAIV prevalence fluctuations. We found seasonal patterns in LPAIV prevalence, with the highest prevalence rates associated to breeding season and autumn migration, matching results from some of the previous studies in Spain and elsewhere, albeit contrasting with others in which the peak of prevalence occurred outside the breeding season [8, 15, 18, 51–54]. We encountered strong interannual longitudinal variation in LPAIV prevalence as in other studies [20]. The results from our model revealed these long-term AIV dynamics to be influenced by a complex process of several ecological and environmental drivers that included the size and composition of the Anseriformes breeding community of the wetland. Thus, results from our model suggest that the waterfowl breeding community composition drives long-term fluctuation of AIV prevalence in our wetland study, similarly to results observed in studies in southern Africa [28, 29], as well as for dabbling ducks in Canada where the proportion of hatch year birds (that ultimately depend on the number of breeding pairs) was positively associated with AIV prevalence [8, 23, 28–31, 52–54]. Bird counts revealed a drastic decrease of breeding couples within the Anseriformes during the course of our study, both at the species and taxa level, while numbers of other resident species increased. The breeding pair decline was most dramatic in the mallard (Figure 1).

Previous studies have emphasised the pivotal role of the Anseriformes in AIV epidemiology, especially the mallard in the Northern Hemisphere [55, 56]. The mallard is the most abundant anatid in the Western Palearctic (its natural distribution range) [57]. This widespread distribution is conferred by its high adaptability towards a wide variety of habitats, ranging from natural ecosystems to anthropic environments [57]. Mallards are social birds capable of moving across long distances during migration, especially subpopulations from high latitudes. However, a proportion of its wild population is migratory whereas another is sedentary [58]. This trait may be involved in different responses towards AIV infections, thus influencing prevalence dynamics [22]. A plausible scenario in AIV epidemiology at the Salburua wetland is that migrants could be responsible for introduction of new AIV strains, while residents may be more important for AIV maintenance. The role of breeders as AIV drivers lies in producing susceptible immunologically naïve offspring, as shown by prevalence peaks reported during autumn migration elsewhere [22, 59].

In fact, significant seasonal differences in AIV prevalence were consistently found in our longitudinal study with regards to host phenology. This finding confirmed the existence of seasonal patterns in the epidemiology of these viruses in wild ecosystems as stated by other authors [14, 18, 20, 51, 56]. AIV prevalence always peaked during the same periods in waterfowl phenology both inter- and intra-annually in the Salburua wetland (Figure 2, Additional files 1 and 3); namely during breeding season and/or during autumn migration, a temporal trend in AIV prevalence that has also been reported previously [56, 60]. However, since our sampling strategy consisted of collecting environmental fresh faecal samples alone, respiratory-tract affinity by potential circulating viral strains during the other periods should not be excluded [51, 61].

During autumn migration, migrating birds of different-origins congregate in large numbers within the same area joining the local avian community, facilitating multiple-strain AIV mixing by host-to-host transmission (within and between species) [18, 62]. Other research groups did not find any relation between AIV detection rates and host density at the species level. However they did when they compared it with the wildfowl community, suggesting aggregation of infection through interspecies mixing reflected in our case study, by the diversity of resident (thus mostly breeding) species [15, 28]. The significant relation of 15 days bs wind with AIV prevalence, also detected by our model, may show the effect of harsh environmental conditions that make the birds remain at the same place rather than scatter, also contributing to host aggregation and locally increased shedding of infectious virus into the environment. AIV detections from wintering populations in the Salburua wetland have been rare, suggesting that prevalence decreases as autumn migration is drawing to an end and more birds have already gained immunity against the circulating viruses [14]. Heterosubtypic immunity may have been protecting the birds against infection from phylogenetically close strains, causing AIV infections to be both less frequent and less diverse during this season and the following spring migration [14, 24]. Nevertheless, AIV has been detected during wintering in Guatemala, The Netherlands, Iran and south central Spain, or during spring migration in Sweden [45, 61, 63–65]. AIV circulation during these periods in some geographical regions could also contribute to year-round virus perpetuation.

Thus, integrating the ecology of both host and LPAIV, a likely scenario is that significantly higher AIV detection rates found during the breeding season and autumn migration are characterised by an input of hosts that are immunologically naïve into the wetland (chicks). The number of offspring is modulated by the number of breeding couples (as a general rule, the more breeding couples the more descendants) and hence, it will have a direct impact on AIV infections (not excluding the emergence of new strains during other periods) [14, 51, 56, 66]. Specifically our dataset reflects a decrease in the Anseriformes breeding pairs, while non-Anseriformes breeding pairs increase. As a consequence, the pool of juvenile susceptible individuals at the end of the breeding season may include a similar total number of individuals, but be composed of a higher number of less susceptible (or less-exposed due to their behaviour) individuals (non-Anseriformes). As a result (in addition to other factors such as heterosubtypic immunity etc.), transmission of AIV could be more limited and progressively lead to reduced prevalence [18, 20, 24]. AIV environmental load, potential persistence and transmission among avian hosts will also be negatively affected contributing to a lower AIV global prevalence. In addition, resident species richness and thus breeding sympatry of different species, has an effect on interspecific transmission of AIV. However, the intrinsic properties of each AIV subtype may modulate these host depending factors.

We observed seasonal and temporal variation in AIV subtype prevalence between sampling periods except for H3N8, which was detected in both sampling periods. This could reflect the influence of heterosubtypic immunity as stated for mallards in empirical and experimental studies [24]. H3N8 was predominant during breeding season while H11 subtypes were only detected during autumn migration. A previous study also found these subtypes mainly during autumn migration but in the case of H3N8 also during breeding season and noticed that H3N8 detection was more consistent at the beginning of autumn migration whereas H11 appeared more frequently at the end of the season [14]. A considerable proportion of the AIV detected belonged to LPAIV H5 or H7 subtypes. Mallards harboured the greatest number of AIV positive cases and subtype richness for both periods (Table 4). H7 subtypes were frequently detected during 2009, all harboured by mallards, although no H7 subtype was detected in mallards in northern Europe between 2008 and 2009 [14]. In contrast, H5 was abundant among findings in the Camargue (France) and Northern European birds during the same period while we only detected a single H5 positive sample (H5N2) [14, 67]. Autumn migration appeared to be the period of the highest subtype richness for both sampling periods, very likely due to a variety of strains brought in by different migrating mallard subpopulations [22].

For AIV surveillance in wild birds, the use of non-invasive sampling techniques such as fresh faeces collection has proven to be a cost-effective tool; large sample sizes can easily be collected from the ecosystem and avian species composition is not discriminated [68]. Capture of birds for swab and blood collection, depending on capture methods, tends to narrow the sample down to specific species and the role of other bird species and potential interspecies transmission in terms of AIV-epidemiology may be missed. Hence, the former sampling strategy gives a more realistic picture of which bird species are being infected and when, avoiding at the same time handling and consequently stressing the animals [68]. The drawbacks are that individual infections, respiratory viral shedding, or previous contact with circulating strains based on antibody detection in sera, cannot be monitored. In this regard, although mallard represented at least 44% of the AIV hosts, it may not necessarily be the only host involved in the epidemiology of the AIV during both sampling periods at this wetland, because other species of ducks, storks and coots were also frequently sampled and to a lower extent gulls, waders and other birds. The low efficiency of the barcoding technique used did not allow the correct host species identification of the remaining AIV positive samples and thus limited our ability to determine the implication of other host species in AIV-epidemiology.

During our sampling-periods no aquatic bird mortality was related to the presence of AIV. In fact, we do not know to what degree the different viral subtypes found during our samplings affect the health status or behaviour of the infected birds. Several studies suggest that LPAIV infections are not pathogenic in their natural reservoir [9, 63]. In any case, this wetland should be regarded as a hotspot for AIV surveillance considering the hazardous potential of the highly diverse LPAIV H5 and H7 subtypes found there.

Understanding the influence of host ecology on pathogen transmission is particularly relevant to prevent and manage wildlife disease emergence [28]. From this perspective, we provide a long-term study on AIV epidemiology in a natural ecosystem where prevalence follows seasonal and annual patterns as previously described, but in which long-term prevalence fluctuation is linked to the Anseriformes breeding community composition and size. The use of non-invasive sampling techniques based on environmental samples has proven effective, although an efficient host-identification tool is still necessary to optimise this sampling strategy.

## Additional files

**Additional file 1.** **Sampling effort (grey bars) and AIV prevalence (orange bars) at each sampling time (between brackets).** The yellow shaded area indicates that during 2010–2011 no samplings were conducted.

**Additional file 2.** **Summary of the stepwise model selection procedure based on Akaike Information Criteria to compare models (AICc) used to model avian influenza virus prevalence.**

**Additional file 3.** **Avian community composition recorded in Salburua wetland.** Mean taxonomic order counts and AIV prevalence according to host phenology. During 2010–2011 birds were counted but no samplings for AIV detection were performed.

## Abbreviations

AIV: avian influenza virus
bs: before sampling
*COI*: mitochondrial cytochrome oxidase subunit 1
GLM: generalized linear model
HA: haemagglutinin
HPAIV: highly pathogenic avian influenza virus
LPAIV: low pathogenicity avian influenza virus
NA: neuraminidase
rRT-PCR: real time reverse transcription polymerase chain reaction
SPF: specific pathogen free
VIF: variance inflation factor
