# Supplementary figures and images for: Long-term avian influenza virus epidemiology in a small Spanish wetland ecosystem is driven by the breeding Anseriformes community
Source: Vet Res. 2019 Jan 17;50:4. doi: 10.1186/s13567-019-0623-5 (PMC6337815; doi:10.1186/s13567-019-0623-5)

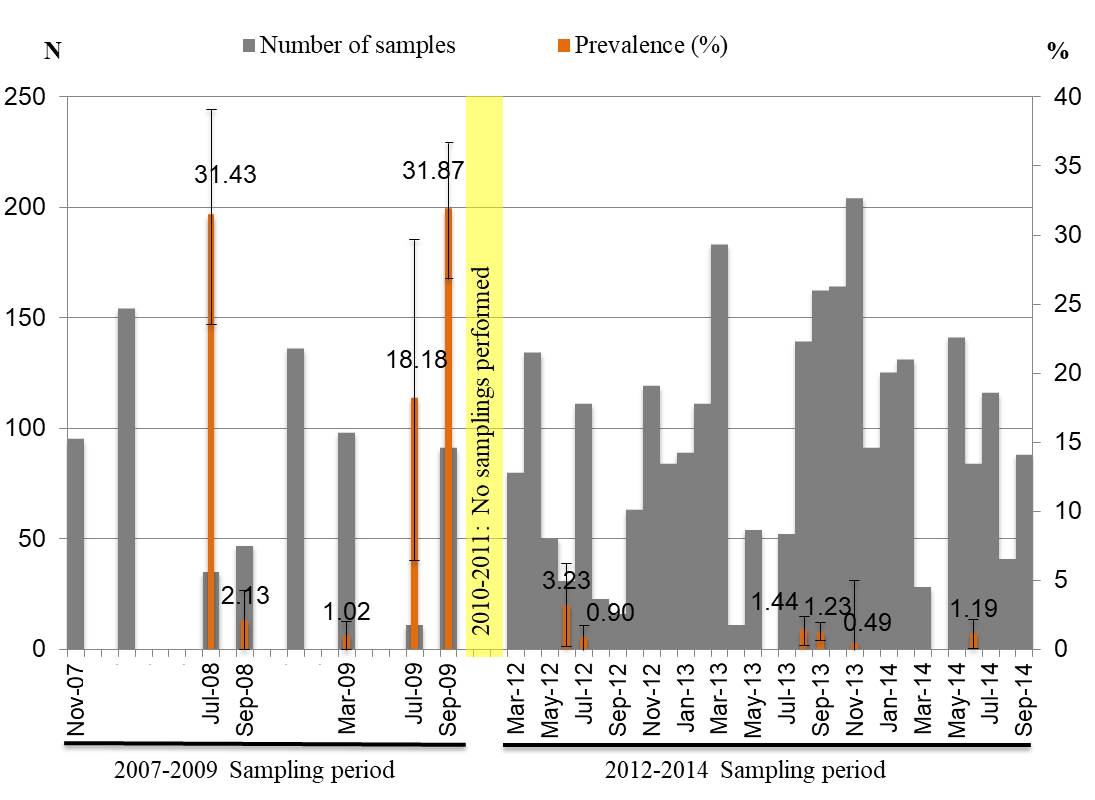

Supplement: Supplementary file 1 — Additional file 1. Sampling effort (grey bars) and AIV prevalence (orange bars) at each sampling time (between brackets). The yellow shaded area indicates that during 2010–2011 no samplings were conducted. [file 13567_2019_623_MOESM1_ESM.jpg]

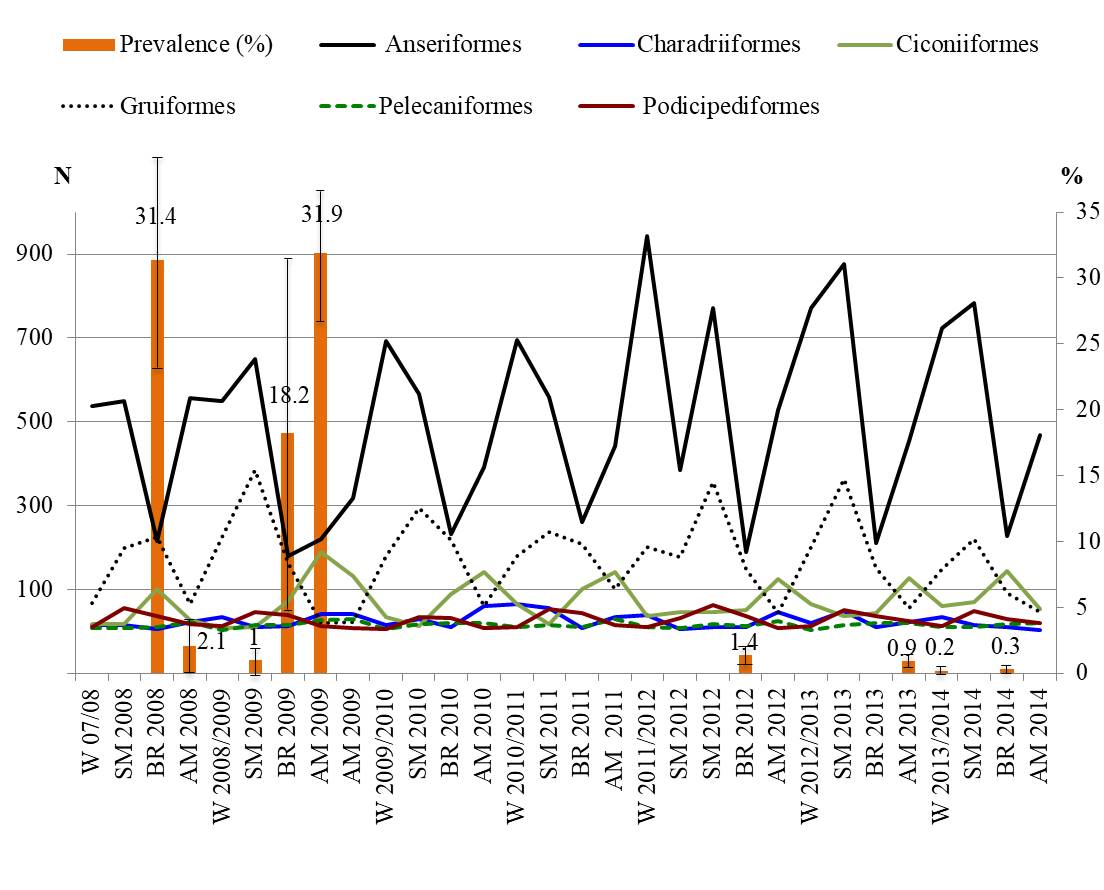

Supplement: Supplementary file 3 — Additional file 3. Avian community composition recorded in Salburua wetland. Mean taxonomic order counts and AIV prevalence according to host phenology. During 2010–2011 birds were counted but no samplings for AIV detection were performed. [file 13567_2019_623_MOESM3_ESM.jpg]
