# Supplementary material for: Long-term avian influenza virus epidemiology in a small Spanish wetland ecosystem is driven by the breeding Anseriformes community
Source: Vet Res. 2019 Jan 17;50:4. doi: 10.1186/s13567-019-0623-5 (PMC6337815; doi:10.1186/s13567-019-0623-5)
Supplement: Supplementary file 2 — Additional file 2. Summary of the stepwise model selection procedure based on Akaike Information Criteria to compare models (AICc) used to model avian influenza virus prevalence. [file 13567_2019_623_MOESM2_ESM.docx]

| **AICc** | **Model specification** | **Code** |
| --- | --- | --- |
| 287.41 | ~1 | Null |
| 170.21 | ~ Breeding Anseriformes couples | V1 |
| 73.08 | ~ V1+Phenology | V2 |
| 68.25 | ~ V2+Resident species richness | V3 |
| 58.28 | ~ V3+Mean wind 15 days before sampling | V4 |
| 54.55 | ~ V4+Breeding *Podiceps cristatus* couples | Final Model |
